# Cardiovascular magnetic resonance in patients with pectus excavatum compared with normal controls

**DOI:** 10.1186/1532-429X-12-73

**Published:** 2010-12-13

**Authors:** Roya S Saleh, J Paul Finn, Michael Fenchel, Abbas Nasirae Moghadam, Mayil Krishnam, Marlon Abrazado, Anthony Ton, Reza Habibi, Eric W Fonkalsrud, Christopher B Cooper

**Affiliations:** 1 Department of Radiology, David Geffen School of Medicine at UCLA, Los Angeles, California, USA; 2 Department of Medicine, David Geffen School of Medicine at UCLA, Los Angeles, California, USA; 3 Department of Biomedical Physics; David Geffen School of Medicine at UCLA, Los Angeles, California, USA; 4 Department of Diagnostic and Interventional Neuroradiology, University of Tuebingen, Germany; 5 Department of Biomedical Engineering, Amirkabir University of Technology (Tehran Polytechnic),Tehran, Iran; 6 Division of Cardiovascular and Thoracic Imaging; Department of Radiology, University of California, Irvine, USA; 7 Exercise Physiology Research Laboratory, Department of Medicine; David Geffen School of Medicine at UCLA, Los Angeles, California, USA; 8 Department of Radiology, Kaiser Permanente, San Diego, California, USA; 9 Department of Radiology, Maricopa Medical Center, Phoenix, Arizona, USA; 10 Department of Surgery, David Geffen School of Medicine at UCLA, Los Angeles, California, USA; 11 Department of Physiology; David Geffen School of Medicine at UCLA, Los Angeles, California, USA

## Abstract

**Purpose:**

To assess cardiothoracic structure and function in patients with pectus excavatum compared with control subjects using cardiovascular magnetic resonance imaging (CMR).

**Method:**

Thirty patients with pectus excavatum deformity (23 men, 7 women, age range: 14-67 years) underwent CMR using 1.5-Tesla scanner (Siemens) and were compared to 25 healthy controls (18 men, 7 women, age range 18-50 years). The CMR protocol included cardiac cine images, pulmonary artery flow quantification, time resolved 3D contrast enhanced MR angiography (CEMRA) and high spatial resolution CEMRA. Chest wall indices including maximum transverse diameter, pectus index (PI), and chest-flatness were measured in all subjects. Left and right ventricular ejection fractions (LVEF, RVEF), ventricular long and short dimensions (LD, SD), mid-ventricle myocardial shortening, pulmonary-systemic circulation time, and pulmonary artery flow were quantified.

**Results:**

In patients with pectus excavatum, the pectus index was 9.3 ± 5.0 versus 2.8 ± 0.4 in controls (P < 0.001). No significant differences between pectus excavatum patients and controls were found in LV ejection fraction, LV myocardial shortening, pulmonary-systemic circulation time or pulmonary flow indices. In pectus excavatum, resting RV ejection fraction was reduced (53.9 ± 9.6 versus 60.5 ± 9.5; P = 0.013), RVSD was reduced (P < 0.05) both at end diastole and systole, RVLD was increased at end diastole (P < 0.05) reflecting geometric distortion of the RV due to sternal compression.

**Conclusion:**

Depression of the sternum in pectus excavatum patients distorts RV geometry. Resting RVEF was reduced by 6% of the control value, suggesting that these geometrical changes may influence myocardial performance. Resting LV function, pulmonary circulation times and pulmonary vascular anatomy and perfusion indices were no different to controls.

## Introduction

Patients with pectus excavatum, a relatively common congenital deformity, often present with symptoms such as exertional dyspnea, fatigue, chest discomfort or palpitations. The suggested etiology for this abnormality, although uncertain, has been described as an overgrowth of the costal cartilages in utero, usually ribs 4, 5, 6, 7, and 8, which then limits the elevation of the sternum [1]. Posterior invagination of the sternum shifts the heart further into the left hemithorax and may distort the shape of the heart and adjacent great veins. Some investigators have reported that the deformed chest is responsible for cardiopulmonary impairment in symptomatic patients. To date, the major evidence for this assertion has been symptomatic improvement after surgery, manifest in enhancement of exercise performance and increased oxygen pulse (a physiological measure that reflects cardiac stroke volume). Haller et al [2] have concluded that corrective surgery improves cardiopulmonary function. Malek et al [3] studied 21 patients and showed the oxygen pulse and maximum oxygen uptake in these patients were significantly lower than reference values. Yalamanchili et al [4] reported a single case of pectus excavatum deformity with extreme leftward displacement of the heart, which compressed the IVC at the level of the diaphragm, causing reduced stroke volume of the right heart. By contrast, some authors have related the cardiopulmonary symptoms to psychological effects in this young group of patients who are hesitant to participate in social events and sports actively [5-7]. Thus, there is no consensus among investigators on the existence or degree of cardiopulmonary impairment in pectus excavatum patients. The clinical significance of the physiological findings in pectus excavatum patients has remained controversial [8,9] leading some to consider corrective surgery a cosmetic procedure.

Currently, radiographic studies such as chest X-ray or CT scan are used to quantify the severity of chest wall deformity by deriving the pectus index (PI). PI, elsewhere called pectus severity index, is defined at the level of greatest deformity as the maximum transverse diameter of the chest divided by the shortest distance between the posterior edge of the indented sternum and the anterior border of the vertebral bodies of the thoracic spine [10,11]. PI is used as a criterion in evaluating patients for surgery, but it has not been shown to have any correlation with stroke volume or even subjective feeling of improvement after correction [12]. On the other hand, the pathophysiology of pectus excavatum patients has been attributed to cardiac displacement as well as the degree and shape of chest deformity and patient age [13].

Chest x-rays and CT scans continue to be used mainly to assess the degree of deformity in pectus excavatum patients but both tests expose patients to ionizing radiation. Other investigations such as pulmonary function tests, maximal exercise tests, arterial blood gases and echocardiography, have also been used to evaluate patients with pectus excavatum. However, they may lack the sensitivity to detect anatomical and subtle pathophysiological abnormalities. Thus, there is a clinical need for additional objective assessment.

Recent technical developments in cardiovascular magnetic resonance (CMR) and the range of its applications have highlighted the potential of this modality as a reliable, non-invasive diagnostic tool. CMR is powerful for the assessment of chest and cardiac anatomy, cardiac function, pulmonary vascular anatomy and perfusion, blood flow and blood circulation times.

To our knowledge, no study has used CMR for assessment of patients with pectus excavatum. The purpose of our study, therefore, was to assess resting cardiovascular anatomy and function in patients with pectus excavatum using CMR and compare the results with healthy control subjects.

## Method and materials

### Study population

Our study population was a group of patients referred or self-referred for evaluation of pectus excavatum. Between August 2004 and January 2008, they were offered the opportunity to participate in a broader study designed to evaluate cardiovascular structure and function using conventional pulmonary function tests and exercise tests as well as imaging. Thirty consecutive patients who enrolled in the broader study and had CMR scans were included in this analysis. The group with pectus excavatum deformity consisted of 23 men and 7 women aged 24.3 ± 13.3 years (age ± SD). Their CMR studies were compared to those from a convenience sample of 25 healthy adult controls within the same age range (18 men, 7 women), aged 24.1 ± 3.8 years, without chest wall deformity or any other cardiovascular or pulmonary disease.

### CMR

#### Apparatus and Setup

All studies were performed on a 32 channel 1.5 Tesla scanner (Magnetom Avanto, Siemens Medical Solutions, Malvern, PA) using combined 24-element spine coil and 6-element body phased array coil. CMR compatible ECG electrodes were positioned on the anterior chest and a 22-gage intravenous cannula was sited in an ante-cubital vein for subsequent infusion of CMR contrast medium. Subjects were positioned supine on the scanner table and advanced head first into the magnet bore. From 2004-2006, the CMR contrast used was gadodiamide 0.15 mmol/kg (GE - Amersham Health Inc. Princeton, NJ) and after this date Magnevist (Berlex Labratories, Wayne, NJ) was used at a lower dose (0.1 mmmole/Kg). This contrast was administered as a controlled, timed infusion using an electronic injector (Spectris, Medrad Inc. Pittsburg, PA).

#### Overview of the CMR Protocol

The following CMR protocol was employed for assessment of cardiopulmonary structure and function. After obtaining scout images, detailed anatomical images were acquired to derive chest wall diameters, pectus severity index, and indices of chest and cardiac distortion. Next, 4-chamber, right and left ventricular outflow tract (RVOT and LVOT) and short-axis cine images were obtained to quantify bi-ventricular function. Dynamic pulmonary parenchymal enhancement and pulmonary-systemic circulation times were then evaluated using low-dose, time-resolved 3D MR angiography with 0.035 mmol/kg gadolinium at a 3D sampling interval of 1.5 seconds. Subsequently, high-spatial-resolution contrast enhanced MR angiography was acquired to visualize detailed pulmonary vascular anatomy. Finally, blood flow in the main pulmonary artery was quantified with velocity-sensitized phase contrast CMR [14]. The individual sequences are outlined below.

#### Anatomical Imaging

A series of large field-of-view (FOV), single-shot, steady-state-free-precession (SSFP) survey images [15] were obtained in coronal, sagittal and transverse planes covering the entire chest and upper abdomen. The following sequence parameters were used: TR/TE = 3.0/1.4 msec and flip angle 70-80°. Accelerated parallel acquisition (generalized autocalibrating partially parallel acquisitions [GRAPPA×2]) [16] was used for enhanced acquisition speed. An average of 40-70 slices (depending on the orientation of the plane and patient size) at 5-6 mm increments were acquired during 14-17 seconds of non-breath hold acquisition.

#### Cine CMR

A stack of short axis cine (SSFP) images (10-12 short adjacent slice positions) were acquired during individual 5-6 second breath-holds, encompassing the atrio-ventricular valve plane to the cardiac apex, followed by cine images in the four-chamber, LV vertical long axis, RVOT, LVOT views and the midline sagittal plane (through the region of minimum sterno-spinal distance). A segmented k-space SSFP sequence as previously described [17] was employed with retrospective ECG-gating. Imaging parameters were TR/TE = 2.8/1.2 msec, flip angle = 70°, GRAPPA×2 and 6 mm slice thickness with a temporal frame duration of 30-43 msec.

#### Dynamic, Time-Resolved 3D MR Angiography

We assessed dynamic pulmonary enhancement as an index of regional lung perfusion using an ultra fast 3D, spoiled GRE sequence [18] with temporal echo sharing [19] (TR/TE 2.5/1, FA 20°). Data acquisition started simultaneously with injection of (6 cc) contrast agent at a rate of 4 ml/sec followed by 20 ml of saline flush with the same rate. 10-14 sequential measurements were acquired at a rate of one frame per 1.5 seconds while subjects held their breath in inspiration. Maximum intensity projection (MIP) reconstruction was performed online and was available for immediate assessment of global vascular anatomy and pulmonary-systemic circulation times.

#### Contrast Enhanced MR Angiography

Dual-phase, high-spatial resolution contrast enhanced MR angiography of the pulmonary vasculature was subsequently acquired using a 3D spoiled gradient-recalled-echo (GRE) sequence (TR/TE: 2.6/0.9 ms, FA: 30°, GRAPPA×3). Gd contrast was infused at a rate of 1.5 ml/sec followed by 30 ml of saline flush at the same rate. A 3D dataset was acquired during 20-22 seconds while subjects held their breath. The entire thorax was interrogated during these acquisitions with voxel dimensions of 1.3 × 1.0 × 1.2 mm³.

#### Velocity Encoded Cine Flow Imaging of the Main Pulmonary Artery

Phase-sensitive velocity encoded cine [20,21] quantified main pulmonary artery flow indices for comparison in both groups. The sequence parameters were TR/TE 55/2.9 ms, FA 20^°^and GRAPPA×2. Images were retrospectively ECG gated with 5 segments per heartbeat and 20 calculated phases. Images were acquired during 18-20 seconds of breath-hold acquisition. The velocity mapping sequence had single direction (through-slice) flow encoding with an aliasing velocity of 150 cm/sec. Two perpendicular RVOT cine images were used to determine the optimal imaging plane. A perpendicular plane to the MPA midway between the pulmonary valve and bifurcation was used.

#### Image Analysis

The MR images were transferred to a 3D workstation (Leonardo, Siemens Medical Solutions, Malvern, PA) for measurement of the following data:

##### Thoracic dimensions

**Figure 1:**
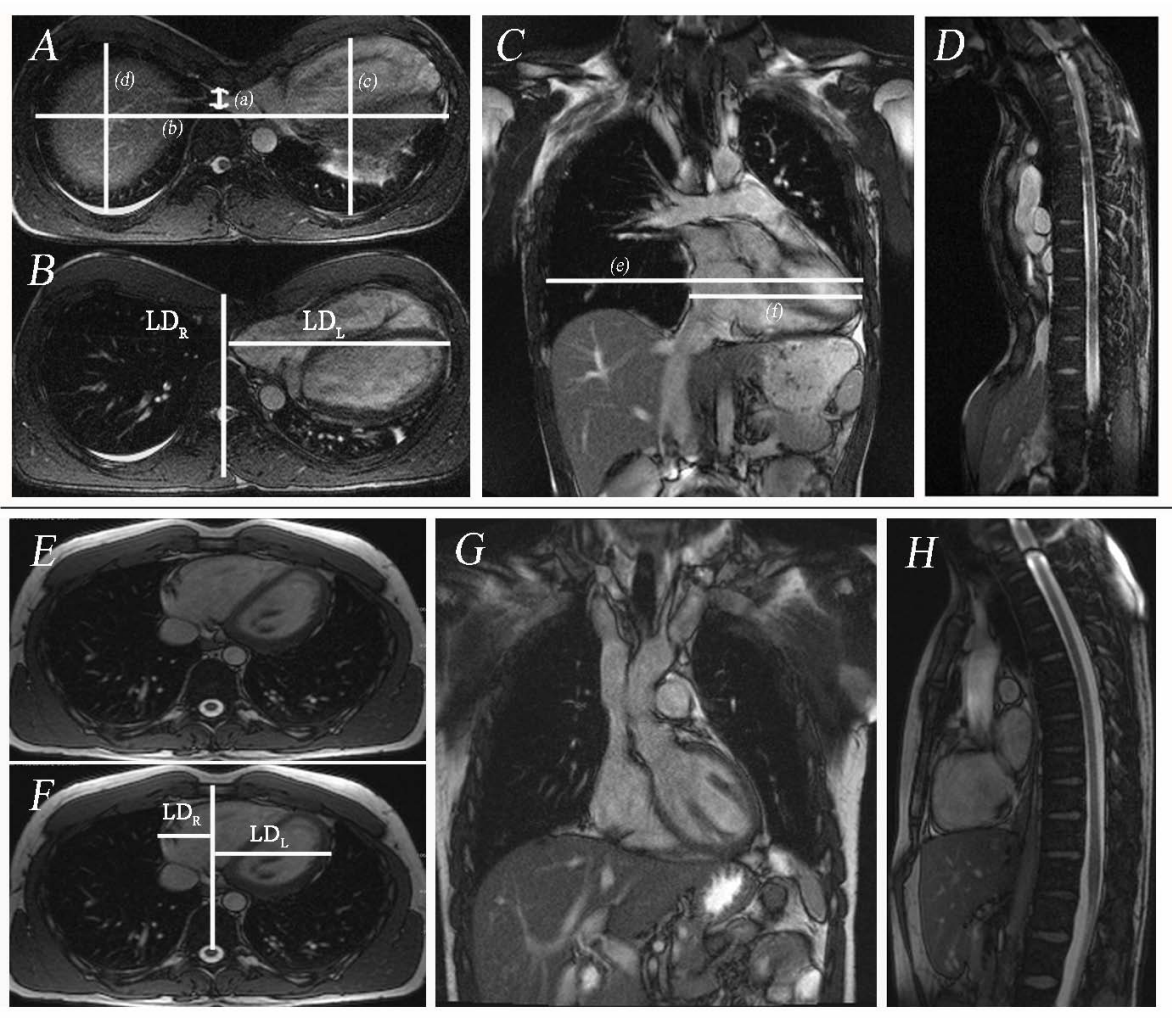
**Chest wall measurements of a 16 year old male with severe pectus excavatum deformity**. **A **Transverse TrueFISP (SSFP) images (TR/TE 630.5/1.4 msec, FA 80°): (a) minimum antero-posterior diameter of the chest (1.2 cm), (b) maximum transverse diameter of the chest (28.2 cm); (c) antero-posterior diameter of the left hemithorax (12.0 cm), (d) antero-posterior diameter of the right hemithorax (11.5 cm). The pectus index (b/a) in this case was 22.9, left chest flatness (b/c) was 2.35 and right chest flatness (b/d) was 2.44. **B **Transverse image showing complete (100%) leftward shift of the heart into left hemithorax. The maximum lateral distances of the left (LD_L _= 15.02 cm) and right (LD_R _= 0 cm) cardiac borders were measured from the midline (sterno-spinal line). Then cardiac left lateral shift (%) was measured as $(\frac{LD_{L}}{LD_{L}+LD_{R}})\times 100$. **C **Coronal TrueFISP image (TR/TE 630.5/1.4; msec, FA 80°) in same patient showing derivation of the indices of cardiac flatness: maximum horizontal diameter of the chest (e) which was 27.8 cm, maximum horizontal diameter of the heart (f) which was 14.8 cm and cardiac flatness (f/e) which was 0.53. **D **Sagittal image showing minimum AP diameter of the chest. **Figure 1E-H **Chest wall measurements in a 23 year old healthy subject male. **E **Transverse TrueFISP (SSFP) images (TR/TE 630.5/1.4; msec, FA 80°): (a) minimum antero-posterior diameter of the chest (9.07 cm), (b) maximum transverse diameter of the chest (24.7 cm); (c) antero-posterior diameter of the left hemithorax (13.3 cm), (d) antero-posterior diameter of the right hemithorax (13.0 cm). The chest index (b/a) in this case was 2.1, left chest flatness (b/c) was 1.8 and right chest flatness (b/d) was 1.9. **F **Transverse image showing leftward shift (67%) of the heart into left hemithorax. The maximum lateral distances of the left and right cardiac borders were measured as: LD_L _= 8.71 cm and LD_R _= 3.97 cm_. _**G and H **are coronal and sagittal TrueFISP images in this healthy subject.

Transverse survey images were interrogated by the lead author who has five years of experience in CMR and data processing, to measure the maximum transverse diameter and minimum sterno-spinal distance, at the level of greatest sternal depression (Figure 1A). The CMR-derived pectus index (chest index) as defined by Raichura et al. [5,22,23] was determined as: $\frac{Max\;transverse\;diameter}{Sternospinal\;dis\tan ce}$. Chest wall flatness was defined using the Nakahara method [10] as the ratio of maximum internal transverse diameter of the thorax to maximum antero-posterior rib diameters on the right and left hemithorax, respectively, at the level of greatest deformity. Additionally, axial images were used to measure the degree of cardiac left-lateral shift. The maximum lateral distances of the left (LD_L_) and right (LD_R _) cardiac borders were measured from the midline (sterno-spinal line). Then cardiac left lateral shift (%) was measured as $\left(\frac{LD_{L}}{LD_{L}+LD_{R}}\right)$. Both LD_L _and LD_R _were measured perpendicular to the sterno-spinal line (Figure 1).

##### Cardiac Function

**Figure 2:**
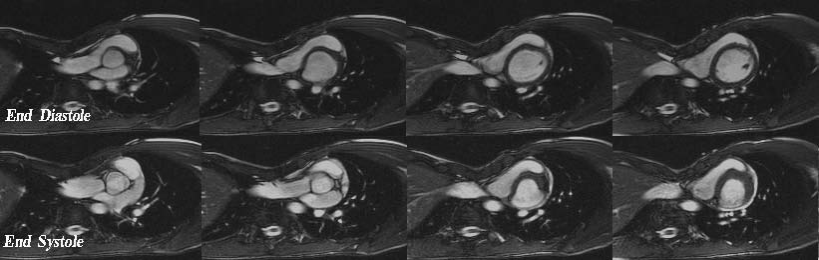
**Cardiac cine images (TR/TE 29.4/1.2, FA 65°) of a 18 year old male with pectus deformity (pectus index being 17.9) and symptoms of exersional dyspnea**. He has also noted discomfort in the lower anterior chest with activity and has experienced tachypnea and tachycardia. The heart is deviated considerably into the left chest by the depressed sternum. Note the restricted dilation at the end-diastolic phase.

Short axis cine images (Figure 2) were assessed by the lead author using commercial cardiac analysis software (Argus, Siemens Medical Solutions) for derivation of cardiac hemodynamic indices. To correct for potential through-plane movement at the cardiac base (because of longitudinal shortening during the cardiac cycle), a manual slice following method was employed. The horizontal long-axis and RVOT were used to display the position of each short-axis cine slice, during the full cardiac cycle. Then images at end-systole (ES) and end-diastole (ED) phases were re-referenced based on the longitudinal shortening of both ventricles. The endocardium and epicardium were traced manually for LV structural assessment whereas only the endocardium was traced for RV volume assessment. End-diastolic volume (EDV), end-systolic volume (ESV), EF, stroke volume (SV) and cardiac output (Qc) were calculated, based on the contoured images. Measurements were normalized to body surface area and used for comparative analysis between patients with pectus excavatum and controls. RV contouring is more subjective and error-prone, so we assessed the possible measurement error in the calculation of RV volumes based on the discrepancy in LV and RV stroke volumes (SV_LV_, SV_RV_) for both groups (SV_LV _- SV_RV_). Furthermore, to evaluate myocardial contractile properties, long and short dimensions (LD and SD) of the right and left ventricles were measured during both ED and ES phases at the level of mid ventricle to calculate regional myocardial shortening ($\frac{ED_{d}-ES_{d}}{ED_{d}}$).

In addition to quantitative measurement of cardiac function, all cine images were assessed qualitatively by two radiologists. Both ventricles were assessed for wall motion abnormalities, aneurysm, chamber enlargement, hypertrophy or wall thinning.

#### Pulmonary-Systemic Circulation Time

**Figure 3:**
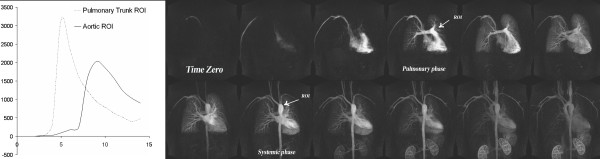
**Pulmonary perfusion in same patient as in figure one**. Coronary time resolved images (TR/TE, FA) show a symmetric pulmonary perfusion with no perfusion deficits and normal cardio-pulmonary transit times in the most severe case among our patients with pectus severity index of 22.9.

We used dynamic time-resolved MRA of the chest to evaluate pulmonary-systemic circulation times and contrast dispersion in the aorta. Francois et al and Shors et al [24,25] showed that cardio-pulmonary circulation times are prolonged in heart disease, and the degree of prolongation is related to the severity of systolic ventricular dysfunction. Therefore, we performed quantitative analysis of pulmonary-systemic circulation times, based on interrogation of the 3D-Time Resolved images using a Mean Curve software algorithm on a commercial workstation (Leonardo, Siemens Medical Solutions). One region of interest (ROI) was placed on the ascending aorta and another on the pulmonary artery trunk. The software outputs the signal versus time curves for both ROIs. Pulmonary-systemic circulation time was defined as the time between peak pulmonary artery enhancement and peak aortic enhancement. Moreover, dispersion of contrast in the aorta was measured as the width of the ascending aorta curve at half its peak signal intensity (FWHM = full width half maximum). (Figure 3)

##### Pulmonary perfusion

Pulmonary parenchymal enhancement was assessed for symmetry and existence of any perfusion deficits by two observers in four anatomical segments (upper and lower lobes in both left and right lungs).

##### Pulmonary Angiographic Anatomy

**Figure 4:**
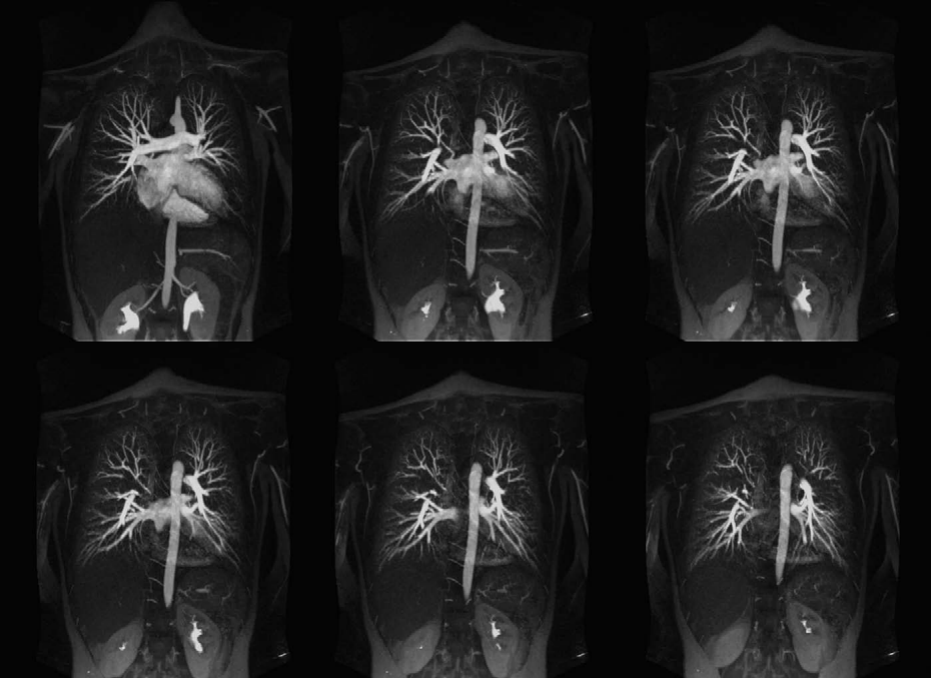
**Contrast enhanced high-resolution MR angiography (TR/TE 2.7/0.9, FA 70°) of a 17-year-old female with a pectus severity index of 7.9 and experience of increasing shortness of breath with exercise**. 15 mm thickness maximum intensity projection images are made with an increment of 1 mm and 9 mm overlap. Images show the normal distribution of the pulmonary vessels with no abnormality. Pulmonary branches are completely assessable up to the 5^th ^branch order.

Assessment of thoracic vascular anatomy was performed by two observers in different sessions, using high-spatial-resolution pulmonary MR angiography and cardiac cine images. Processing was performed on a 3D workstation (Leonardo, Siemens Medical Solutions, Malvern, PA), using a maximum intensity projection (MIP) algorithm. The 3D volume source images were reconstructed into overlapping thin (10 mm) MIP sub-volumes (Figure 4). Pulmonary trunk, main and lobar arteries on MRA images and MIP reconstructed images were assessed for existence of any degree of abnormalities (e.g. aneurysm, stenosis).

##### Flow Measurement in the Main Pulmonary Artery

In the main pulmonary artery, the following measurements were performed: peak and average velocity, peak and average volume flow (as product of mean or peak blood velocity by cross-sectional area), and net forward volumes (integrated forward volume flow) [14].

### Statistical Analysis

A two-tailed t-test was used to compare chest indices, cardiac indices, pulmonary flow measurements and pulmonary-systemic circulation times between patients with pectus excavatum and controls. In addition, the Kolmogrorov-Smironov Test was used to compare the distribution of measurement error for left and right ventricular EF and RV_sv_. Chest indices were correlated with ventricular EF using Pearson correlation. A correlation coefficient (r) greater than 0.6 was considered to be a strong correlation. Inter-observer agreement between readers for the assessment of thoracic structure and cardiac function was determined by calculating the kappa coefficient. (poor agreement, κ = 0; slight agreement, κ = 0.01-0.2; fair agreement, κ = 0.21-0.4; moderate agreement, κ = 0.41-0.6; good agreement, κ = 0.61-0.8; and excellent agreement, κ = 0.81-1) [26,27]. At all comparisons, P < 0.05 was considered to indicate a statistically significant difference. Data analysis was performed using SPSS 13, 2004 (SPSS Inc, Chicago, IL) statistical package. The chest indices, cardiac indices, pulmonary flow measurements, and pulmonary-systemic circulation times were plotted as mean ± SD.

### Ethical Considerations

This HIPAA compliant study was approved by the Institutional Review Board of the David Geffen School of Medicine, University of California, Los Angeles. Written informed consent was obtained for all study subjects.

## Results

### Chest wall measurements and indices

Chest wall measurements and all indices are summarized in Table 1. As expected, there was a significantly lower minimum sterno-spinal distance in pectus excavatum patients (3.6 ± 1.7 cm) compared with controls (9.1 ± 1.6 cm; P < 0.001). Right and left hemithorax maximum AP rib diameters were also lower than controls (P < 0.01). The chest wall index was three times higher in pectus excavatum patients (mean ± SD in pectus excavatum patients 9.3 ± 5.0, ranging from mild (3.7) to a very severe deformity (22.9) versus 2.8 ± 0.4 in the control group ranging from 2.1 to 3.9 (P < 0.001). The inclusion criteria for the major study stipulate a chest wall index or pectus index (PI) >3.5. Likewise, chest flatness was significantly greater in pectus excavatum patients (2.2 ± 0.2 and 2.1 ± 0.2 for right and left CF respectively) than in controls (1.7 ± 0.2 and 1.8 ± 0.2 for right and left CF respectively, P < 0.001). Cardiac left lateral shift (%) was significantly higher in pectus excavatum patients (83.9% ± 13.6) compared to the controls (65.2% ± 5.8; P < 0.001).

**Table 1:** Summary of chest and cardiac dimensions and indices

	Pectus patients	Healthy Subjects	
	
	Mean ± SD	Mean ± SD	P-value
Age (year)	24.3 ± 13.3	24.1 ± 3.8	0.770
Height (meter)	1.8 ± 0.1	1.7 ± 0.07	0.253
Weight (Kg)	67.1 ± 11.7	72.2 ± 14	0.054
BSA	1.8 ± 0.2	1.9 ± 0.2	0.055

**Chest Diameters and Indices**			

Minimum sterno-spinal distance (cm)	3.6 ± 1.7	9.1 ± 1.6	**< 0.001**
Maximum transverse diameter (cm)	26.4 ± 2.1	25.1 ± 2.0	0.057
Chest Index/Pectus Severity Index (PSI)	9.3 ± 5.0	2.8 ± 0.4	**< 0.001**
Right chest maximum AP diameter (cm)	12.2 ± 1.6	14.5 ± 1.7	**< 0.001**
Left chest maximum AP diameter (cm)	12.6 ± 1.6	14.2 ± 2.0	**0.003**
Right chest flatness	2.2 ± 0.2	1.7 ± 0.2	**< 0.001**
Left chest flatness	2.1 ± 0.2	1.8 ± 0.2	**< 0.001**
Coronal maximum transverse diameter (cm)	25.7 ± 3.2	24.3 ± 1.0	0.072
Coronal maximum diameter of the heart (cm)	14.4 ± 2.3	12.8 ± 1.4	**< 0.001**
Cardio-Thoracic Ratio (Cardiac Flatness)	0.6 ± 0.3	0.5 ± 0.04	0.154

**Cardiac Shift**			

Right Border of heart to midline (cm)	2.7 ± 2.1	4.3 ± 0.7	**< 0.001**
Left Border of heart to midline (cm)	11.6 ± 1.6	8.1 ± 1.3	**< 0.001**
Cardiac Left Lateral Shift (%)	83.9 ± 13.6	65.2 ± 5.8	**< 0.001**

### Cardiac Hemodynamic Indices

Quantitative cardiac functional indices, mean ± SD, both absolute and normalized to body surface area (BSA), are presented in Table 2. Both left- and right-ventricular ejection fractions (LVEF and RVEF) were lower in patients compared to controls. RVEF achieved a statistically significant difference of approximately 6% (mean ± SD in pectus excavatum patients 53.9 ± 9.6 versus 60.5 ± 9.5% in controls P = 0.013) when compared to the control group. Furthermore, a Kolmogorov-Smirnov Test used to compare the distribution of measurement error for both groups, failed to show a significant difference (P > 0.05). RV End-systolic volume (ESV) was significantly higher in pectus excavatum patients (mean of normalized value ± SD for pectus excavatum patients were 30.8 ± 10.5 versus 23.9 ± 7.5 ml in controls; P = 0.006). Measurement error between both groups was not significantly different from each other (P > 0.05).

**Table 2:** Cardiac Hemodynamic Indices

			Pectus patients		Healthy Subjects		
			**Mean ± SD**	**Range**	**Mean ± SD**	**Range**	**P-value**

**Left Ventricle**	ABSOLUTE	EF (%)	66.1 ± 6.6	56.3 - 82.6	69.7 ± 6.9	54.4 ± 82.5	0.059
		EDV (mL)	113.5 ± 22.8	67.6 - 164.7	111.4 ± 29.9	56.4 ± 202.3	0.620
		ESV (mL)	38.4 ± 10.9	13.7 - 59.6	34.3 ± 15.2	15.8 ± 92.4	0.182
		Myocardial Mass (g)	122.2 ± 32.1	66.4 - 211.3	117.9 ± 34.4	25.6 ± 188.2	0.287
		SV (mL)	75.0 ± 16.1	43.5 - 110.2	76.9 ± 19.1	37.1 ± 115.9	0.782
		CO (l/min)	5.03 ± 1.3	3.2 - 9.3	4.8 ± 1.1	2.5 ± 6.5	0.427
	
	NORMALIZED (units/m^2)	EDV (mL)	61.7 ± 12	40.5 - 81.9	60.2 ± 13.7	37.2 ± 95.9	0.564
		ESV (mL)	21.4 ± 6.4	7.0 - 34.4	18.5 ± 7.2	7.7 ± 43.8	0.127
		Myocardial Mass (g)	65.8 ± 13.5	38.2 - 91.7	60.2 ± 17.5	13.9 ± 89.2	0.602
		SV (mL)	40.8 ± 7.7	24.7 - 61.5	41.7 ± 9.1	24.4 ± 63.7	0.703
		CI	2.7 ± 0.6	1.85 - 4.01	2.6 ± 0.6	1.6 ± 3.7	0.421

**Right Ventricle**	ABSOLUTE	EF (%)	53.9 ± 9.6	33.9 - 73.0	60.5 ± 9.5	42.3 ± 80.8	**0.013**
		EDV (mL)	122.1 ± 30.5	63.8 - 168.6	114.9 ± 36.4	57.1 ± 240.0	0.370
		ESV (mL)	56.5 ± 18.8	28.8 - 98.1	44.6 ± 17.2	23.3 ± 108.5	**0.014**
		SV (mL)	65.6 ± 19.3	27.9 - 103.9	69.9 ± 24.8	21.7 ± 138.4	0.555
		CO (l/min)	4.3 ± 1.3	2.3 - 8.6	4.3 ± 1.5	1.4 ± 8.2	0.896
	
	NORMALIZED (units/m^2)	EDV (mL)	66.3 ± 16.2	36.7 - 97.1	61.9 ± 16.9	34.3 ± 115.1	0.306
		ESV (mL)	30.8 ± 10.5	14.3 - 54.3	23.9 ± 7.5	11.4 ± 51.5	**0.006**
		SV (mL)	35.4 ± 9.8	16 - 58.8	37.9 ± 12.8	14.3 ± 65.7	0.465
		CI	2.3 ± 0.6	1.2 - 3.6	2.3 ± 0.8	0.9 ± 4.4	0.987

EF = Ejection Fraction; EDV = End Diastolic Volume; ESV = End Systolic Volume; SV = Stroke Volume; CO = Cardiac Output; CI = Cardiac Index

**Figure 5:**
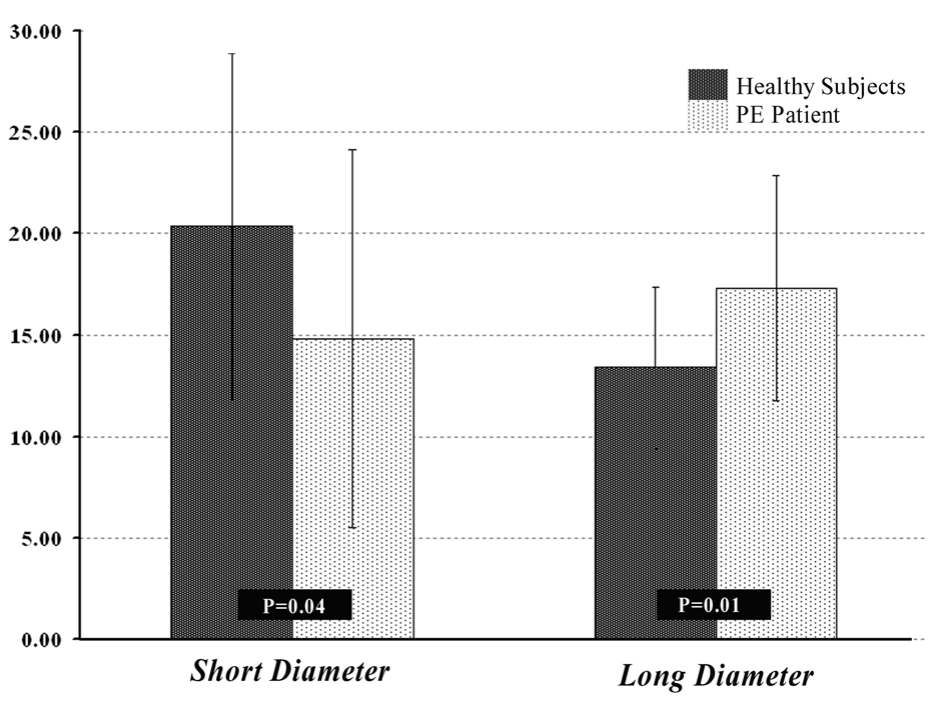
**Comparison of right ventricle myocardial shortening in the direction of short and long diameters between patients with pectus excavatum and healthy control subjects**.

The RV short dimension was significantly less both at end-diastole (ED) (pectus excavatum versus control: 22.6 ± 7.0 mm and 33.6 ± 4.6 mm, P < 0.001) and end-systole (ES) (18.9 ± 5.9 mm versus 26.7 ± 4.3 mm, P < 0.002). The RV long dimension was significantly greater at ED (79.1 ± 9.6 mm versus 73.3 ± 9.0 mm for pectus excavatum patients and controls respectively; P = 0.047). The short diameter RV shortening was significantly less in pectus excavatum patients (pectus excavatum versus control: 14.8 ± 9.3 versus 20.4 ± 8.5, P = 0.043) and the long diameter RV shortening was significantly greater in pectus excavatum patients (pectus excavatum versus control: 17.4 ± 5.5 versus 13.4 ± 3.9, P = 0.007) (Figure 5). However, we did not detect any significant differences in these measures at ES (P > 0.512). We did not detect any significant differences for either LV short and long dimensions nor in their myocardial shortening (P > 0.2) Table 3.

**Table 3:** Ventricular diameters and shortening indices (mean ± SD) based on mid-ventricle short-axis cine images

		Pectus patients	Healthy Subjects	P-value
**Ventricular Diameters during cardiac phases (mm)**				

**RV-SD**	ED	22.6 ± 7.0	33.6 ± 4.6	**< 0.001**
	ES	18.9 ± 5.9	26.7 ± 4.3	**< 0.002**
**RV-LD**	ED	79.1 ± 9.6	73.3 ± 9.0	**0.047**
	ES	65.4 ± 9.0	63.6 ± 8.9	0.512

**LV-SD**	ED	45.6 ± 5.9	46.6 ± 4.7	0.517
	ES	30.2 ± 4.5	31.67 ± 4.1	0.259
**LV-LD**	ED	54.7 ± 6.4	55.4 ± 3.9	0.618
	ES	35.8 ± 4.8	36.0 ± 4.7	0.857

**Myocardial Shortening (%)**				

RV-SD shortening		14.8 ± 9.3	20.4 ± 8.5	**0.043**
RV-LD shortening		17.4 ± 5.5	13.4 ± 3.9	**0.007**
LV-SD shortening		33.6 ± 6.6	32.0 ± 5.9	0.404
LV-LD shortening		34.3 ± 6.7	34.9 ± 6.9	0.766

RV-SD = right ventricle short diameter; RV-LD = right ventricle long diameter; LV-SD = left ventricle short diameter; LV-LD = left ventricle long diameter; ED = end diastole; ES = end systole

We did not detect any significant correlation between the RVEF or LVEF and pectus index. (For RVEF r = -0.2061 (95% confidence interval -0.5272 to 0.1666), P = 0.2746; For LVEF r = -0.0065 (95% confidence interval -0.3659 to 0.3546) P = 0.9727).

### Pulmonary Perfusion and Pulmonary-Systemic Circulation Time

All dynamic 3-D MR angiographic studies showed symmetrical perfusion in both lungs, with no filling defects. There was no difference between both readers for pulmonary perfusion assessments.

Pulmonary-systemic circulation times in pectus excavatum patients were not significantly different (mean ± SD for pulmonary-systemic circulation time and FWHM in pectus excavatum patients was 6.3 ± 1.1 and 6.7 ± 1.0 seconds respectively, versus 6.6 ± 0.5 and 6.5 ± 1.1 seconds in controls; for both P > 0.2).

### Pulmonary Vascular Anatomy

One patient had an enlarged pulmonary artery trunk (3.2 cm), detected by both readers but no other vascular abnormality. Another patient had dextrocardia but no evidence of valvular or cardiac septal abnormality. Observing that this patient did have cardiac compression caused by the pectus deformity, we chose to not to exclude him/her from the overall analysis. The remaining patients were diagnosed with normal pulmonary vascular anatomy by both readers independently (κ = 1).

### Pulmonary Artery Flow Indices

Average flow and net forward volume indexed to BSA tended to be greater in patients; however, the differences failed to reach statistical significance (P = 0.070 and P = 0.386 respectively). The results showed no significant difference for all other flow parameters (P > 0.1). Quantitative pulmonary artery flow measurements for both patients and controls are summarized in Table 4.

**Table 4:** Pulmonary trunk assessment for dimensions and flow measurements (mean ± SD)

	Pectus Patients	Healthy Subjects	P-value
Pulmonary Artery Diameter (cm)	2.18 ± 0.2	2.13 ± 0.1	0.574
Average Cross-sectional Area (cm^2^)	6.2 ± 1.0	6.1 ± 0.9	0.623
Average Velocity (cm/sec)	16.5 ± 4.4	15.0 ± 2.0	0.175
Peak Velocity (cm/sec)	78.9 ± 21.0	75.2 ± 14.0	0.542
Average Flow (ml/m)	101.0 ± 16.0	89.0 ± 15.6	0.070
Average Flow/min (l/min)	5.9 ± 0.9	5.3 ± 0.9	0.121
Forward Volume (ml)	86.1 ± 18.3	79.6 ± 18.1	0.405
Reverse Volume (ml)	0.2 ± 0.5	0.2 ± 0.3	0.615
Net Forward Volume (ml)	86.1 ± 18.9	79.4 ± 17.9	0.386
Net Forward Volume/BSA (ml/m^2^)	47.3 ± 10.7	43.4 ± 7.6	0.299

## Discussion

The results of our study show that, compared with controls, patients with pectus excavatum deformity have reduced RV short axis diameter, increased RV long axis diameter and reduced RV ejection fraction. These findings likely reflect compression induced changes in RV anatomy and changes in the pattern of myocardial shortening in patients with pectus excavatum, potentially mimicking restrictive RV physiology. No differences were found in LV ejection fraction, LV myocardial shortening, aorto-pulmonary circulation time or pulmonary flow indices between pectus excavatum patients and controls.

Most of our patients presented with complaints of increasing dyspnea with mild exercise or a decrease in stamina and endurance. Some physicians have suggested that these symptoms and their improvement after corrective surgery are, at least in part, psychological [5,6]. Others have demonstrated that patients with pectus excavatum have true physiological impairments [3] and some studies report increased exercise tolerance and higher oxygen pulse after corrective surgery [2]. In our study, patients with pectus excavatum had slightly lower RVEF compared to the healthy control group, which did not show any significant difference in their distribution from the control group. While CMR can provide a very good visualization and measurement of LV volumes, RV volume measurements are more challenging. RV contouring is more challenging, because of the thinner wall, widespread trabeculation and geometrically non-uniform shape. RVOT imaging becomes even more difficult when the RV is distorted by sternal compression and this can exacerbate error in RV volume measurement. Nevertheless, we found same systematic error of stroke volume discrepancy in LV and RV between both groups (P = 0.510). The reduced RVEF could be explained by the higher ESV in pectus excavatum patients, implying altered myocardial contractility rather than RV restriction by direct sternal depression. The RV wall is very thin and most of the myocardial thickening occurs in the trabeculated portion [28]. If this portion is incompletely segmented from the blood pool, the measured end systolic blood volume will be increased. Meticulous contouring is therefore, important to minimize errors in measurement.

Our results show that RV myocardial shortening was significantly different in patients with pectus excavatum compared with controls. Myocardial shortening and thus contractility in the direction of the short-diameter (SD) in short axis cine was reduced, whereas myocardial shortening in the direction of the long-diameter (LD) was increased. It appears that this is a compensatory mechanism in order to preserve RVEF.

We did not detect any significant correlation between the EF and pectus severity index, which could potentially be explained by chest reconfiguration (i.e. an increase in maximum transverse diameter of the chest) and cardiac left lateral shift, which could be another compensatory mechanism to avoid the direct restrictive impairment of the right heart. Furthermore, pectus excavatum patients did not show significant changes in pulmonary flow quantification.

### Limitations

Our study has certain limitations. Firstly, it was not strictly a case-control design and the normal comparison group was only matched in terms of age range. They were, however, healthy volunteers without any history of cardiovascular or lung disease and without any chest wall deformity. Furthermore, we detected no abnormalities on CMR scanning in this group. Secondly, while CMR can provide a very good visualization and measurement of LV volumes, RV contouring and volume measurements can be more challenging. This is because of the thinner wall, widespread trabeculation and geometrically non-uniform shape. RVOT imaging becomes even more difficult when the RV is distorted by sternal compression and this can exacerbate error in RV volume measurement. Finally, patients with severe pectus excavatum deformity (or even moderate pectus excavatum deformity) may be different from the mild forms in terms of cardiopulmonary function as quoted by Haller et al [2]. Unfortunately, given the relatively small numbers of subjects, especially with very severe pectus excavatum, we were unable to stratify patients according to the severity of pectus excavatum. We did, however look for correlations between PI and both LVEF and RVEF but found none. However, this could reflect the fact that PI is not such a satisfactory means of assessing the severity of pectus excavatum and that other measures reported in this paper might be more promising for future research investigations.

## Conclusion

Patients with pectus excavatum deformity show varying levels of distortion of the right ventricle, associated with a 6% reduction in resting RVEF relative to controls. The degree of physiological impairment is likely related to the degree of geometrical distortion of the RV due to the sternal compression, and it is possible that, in some patients, restrictive physiology plays a role. Further work is warranted to assess whether these changes are reversible with corrective surgery.

## Competing interests

Dr. Christopher Cooper is the recipient of the grant. The rest of the authors declare that they have no competing interests'.

## Authors' contributions

RS: Coordinated the project, recruited volunteer subjects, performed MRI scans in volunteer subjects, performed quantitative evaluation of MRI images, performed literature review, statistical analysis and drafted the manuscript. JPF: Conceived of the comparative study design between patients and the control group, participated in the design of the study, prepared the MRI protocol for the study and participated in manuscript preparation. MF: Performed literature review and data interpretation. AM: Performed data analysis and helped to draft the manuscript. MK: Performed qualitative evaluation of MRI images for both healthy subjects and patient group. MA: Recruited study subjects, gathered subject demographic and medical history data, coordinated MRI scan schedule. AT: Performed qualitative evaluation of MRI images for both healthy subjects and patient group. RH: Performed image and data processing, participated in statistical analysis of data. EWF Participated in the study design and helped to draft the manuscript. CBC: Conceived of the study, participated in the study design, and participated in manuscript preparation. All authors participated in manuscript editing. All authors read and approved the final manuscript.
